# The Monoamine Re-Uptake Inhibitor UWA-101 Improves Motor Fluctuations in the MPTP-Lesioned Common Marmoset

**DOI:** 10.1371/journal.pone.0045587

**Published:** 2012-09-20

**Authors:** Philippe Huot, Tom H. Johnston, Michael N. Gandy, M. Gabriela Reyes, Susan H. Fox, Matthew J. Piggott, Jonathan M Brotchie

**Affiliations:** 1 Toronto Western Research Institute, University Health Network, Toronto, Ontario, Canada; 2 Division of Neurology, Movement Disorder Clinic, Toronto Western Hospital, University Health Network, Toronto, Ontario, Canada; 3 School of Chemistry and Biochemistry, The University of Western Australia, Perth, Australia; INSERM/CNRS, France

## Abstract

**Background:**

The wearing-OFF phenomenon is a common motor complication of chronic L-3,4-dihydroxyphenylalanine (L-DOPA) therapy for Parkinson’s disease. We recently described the discovery of UWA-101, a dual serotonin (SERT) and dopamine (DAT) transporter inhibitor, which increases the duration of “good quality” ON-time provided by L-DOPA in the 1-methyl-4-phenyl-1,2,3,6-tetrahydropyridine (MPTP)-lesioned primate. Here, we further characterise the effects of UWA-101 on this extension of ON-time in terms of L-DOPA-induced side-effects in the MPTP-lesioned common marmoset.

**Methods:**

Marmosets were rendered parkinsonian by MPTP injection and “primed” by repeated L-DOPA administration, to exhibit dyskinesia and psychosis-like behaviours. Animals were then administered acute challenges of L-DOPA in combination with UWA-101 (1, 3, 6 and 10 mg/kg) or vehicle.

**Results:**

In combination with L-DOPA, UWA-101 (3, 6 and 10 mg/kg) significantly increased duration of ON-time (by 28%, 28%, and 33%, respectively; all *P*<0.05). UWA-101 (10 mg/kg) significantly extended duration of ON-time without disabling dyskinesia (by 62%, *P*<0.01). UWA-101 did not exacerbate the severity of dyskinesia (*P*>0.05). However, at the highest doses (6 and 10 mg/kg), UWA-101 increased the severity of psychosis-like behaviours (*P*<0.05).

**Conclusions:**

Our results demonstrate that dual SERT/ DAT inhibitors can effectively enhance L-DOPA anti-parkinsonian action, without exacerbating dyskinesia and, as such, represent a promising new therapeutic class for wearing-OFF. However, at higher doses, dual SERT/ DAT inhibitors may exacerbate dopaminergic psychosis.

## Introduction

The cardinal manifestations of Parkinson’s disease (PD) are caused by the degeneration of dopaminergic neurons of the substantia nigra, which leads to a deficit of dopamine in the striatum [1]. Dopamine replacement therapy with L-3,4-dihydroxyphenylalanine (L-DOPA) is the most effective treatment for PD [2]. However, long-term treatment with L-DOPA is associated with motor and non-motor complications, such as dyskinesia, wearing-OFF and hallucinations [3].

The wearing-OFF phenomenon can be described as a shortening in the duration of anti-parkinsonian benefit, ON-time. Wearing-OFF typically begins after a few years of L-DOPA treatment, affecting 41% of patients after 5 years [4] and more than 90% of PD patients after 15 years of dopaminergic therapy [3]. Currently, the drugs available clinically to extend duration of L-DOPA anti-parkinsonian action are either catechol-O-methyltransferase (COMT) inhibitors, such as entacapone and tolcapone, or monoamine oxidase type B (MAO-B) inhibitors, such as selegiline and rasagiline. However, the efficacy of these classes of drug in extending duration of daily ON-time may be only modest. For instance, in the LARGO [5] and PRESTO [6] studies, rasagiline increased ON-time duration by 12–21%, while entacapone extended ON-time duration by 21%. Moreover, the benefit of these classes of drugs can be compromised by their potential to worsen severity and/or proportion of ON-time affected by dyskinesia. Thus, entacapone has been shown to effectively enhance duration of ON-time and reduce duration of OFF-time, but also significantly increase dyskinesia severity [7]. Reducing the L-DOPA dose can reduce dyskinesia, but at the expense of worsening parkinsonism. Thus, there is a need to develop drugs with the potential to increase ON-time duration without exacerbating dyskinesia *i.e*. “good quality” ON-time.

Monoamine re-uptake inhibitors block the dopamine, serotonin, or noradrenaline transporters (DAT, SERT and NET, respectively), thereby having potential to increase synaptic levels of these transmitters. With respect to PD, DAT inhibition could prolong the time dopamine remains in the synaptic cleft and thus the time it exerts its biological effects, thereby, at least in theory, extending ON-time. Monoamine re-uptake inhibitors with different selectivity for DAT over the other transporters have been assessed in PD, with varying efficacy. Thus, the non-selective triple monoamine re-uptake inhibitor tesofensine, in combination with L-DOPA, failed to improve motor function in PD in one study [8], had a non-sustained effect in another [9], and significantly decreased daily OFF-time duration in a third [10]. Brasofensine, a dual DAT/ NET inhibitor, was an effective anti-parkinsonian agent as monotherapy in the 1-methyl-4-phenyl-1,2,3,6-tetrahydropyridine (MPTP)-lesioned common marmoset, but did not enhance L-DOPA anti-parkinsonian action when administered as adjunct therapy [11]. Consistent with these findings the combination of brasofensine with L-DOPA did not improve L-DOPA anti-parkinsonian action in a clinical study [12].

Thus, while monoamine re-uptake inhibitors may have potential as therapeutic agents in PD, the drug candidates identified so far may not possess ideal combinations of DAT activity relative to SERT or NET to enhance the actions of L-DOPA. We have recently described the discovery of a novel monoamine re-uptake inhibitor, UWA-101 (*N*-methyl-1-cyclopropyl-1-piperonylmethylamine (2-(benzo[*d*] [1], [3]dioxol-5-yl)-1-cyclopropyl-*N*-methylethanamine)) [13]. To our knowledge, UWA-101 is the first dual, essentially equipotent, SERT/ DAT inhibitor to be described as showing efficacy in pre-clinical models, as an adjunct to clinically-relevant doses of L-DOPA. Specifically, when administered with L-DOPA, UWA-101, increased the proportion of ON-time that was not compromised by disabling dyskinesia in the MPTP-lesioned marmoset [13]. However, this initial study was not designed to determine whether UWA-101 could extend the total duration of ON-time, nor the impact of UWA-101 on psychosis-like behaviours, which, like dyskinesia, may be a significant problem in the treatment of PD [14].

The present study thus examined the effects of a wider range of dose of UWA-101, employed a longer period of assessment to enable characterisation of the duration, as well as quality of the extended ON-time, and to assess the effect of UWA-101 on psychosis-like behaviours.

## Materials and Methods

### UWA-101 Synthesis

**Figure 1:**
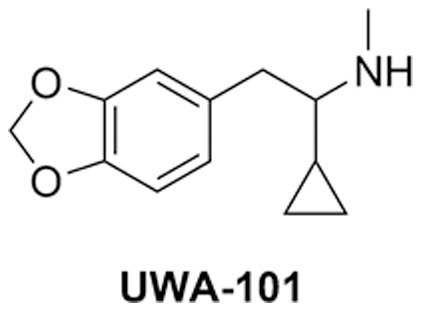
Chemical structure of UWA-101.

UWA-101 (Figure 1) was synthesised by reductive amination of piperonyl cyclopropyl ketone with methylamine as described previously [15]. UWA-101 was converted to its water soluble, crystalline hydrochloride and tested as such.

### Behavioural Assessment of UWA-101 in the MPTP-lesioned Common Marmoset

#### Ethics statement

Five female common marmosets (*Callithrix jacchus*, 300–500 g; Harlan, Madison, USA) were kept under conditions of controlled temperature (25±2°C) and lighting (12 h light/ dark cycle, 08:00 lights on). Animals were cared for in accordance with an IACUC approved by University Health Network Animal Care Committee protocol and with the regulations defined by the Canadian Council on Animal Care. Animals were housed in groups of 2–3 and had unrestricted access to food, fresh fruit supplements and water, and their home cage was enriched with primate toys, perches and auditory stimuli. Efforts were made to reduce to a minimum the number of animals required for statistically valid analyses and to minimise their suffering. Prior to the start of studies, animals were acclimatised to handling, administration of subcutaneous (s.c.) treatments, and transfer to observation cages.

### Induction of Parkinsonism and Dyskinesia in the Common Marmoset

Animals were rendered parkinsonian by administration of MPTP hydrochloride (2 mg/kg s.c. daily, for 5 d; Sigma, St Louis, USA). Following MPTP treatment, marmosets entered a 12 week recovery period to allow parkinsonian symptoms to develop and stabilise. Treatment-related complications, including dyskinesia and psychosis-like behaviours, were induced by administration of oral Prolopa® (L-DOPA/ benserazide 15/3.75 mg/kg twice daily; Hoffmann-La Roche Limited, Mississauga, Canada) for a minimum of 30 d. This treatment regimen has been demonstrated to produce a stable model of L-DOPA-induced motor and non-motor complications [16], [17]. The animals used in the present experiment had not been used in previous studies and were drug-naïve, with the exception of L-DOPA.

### Administration of UWA-101 in Combination with L-DOPA to the Parkinsonian Marmoset

On days of behavioural assessment, marmosets were administered L-DOPA/ benserazide 25/6.25 mg/kg s.c. (Sigma-Aldrich, St Louis, USA) in combination with either vehicle (NaCl 0.9%) or UWA-101 (1, 3, 6, 10 mg/kg of drug free base) s.c. at 09:00. The drug administration schedule was fully randomised between and within animals, according to a Latin square design (Experimental Design Generator And Randomiser (EDGAR), http://www.edgarweb.org.uk/), in which all animals received all treatments. Immediately after treatment administration, marmosets were placed individually into observation cages (0.8 × 0.8 × 0.7 m) containing food, water and a wooden perch, and were left undisturbed for the 6 h duration of the experiment. Behaviour was recorded via DVD footage for *post hoc* analysis by a neurologist specialised in movement disorders blinded to the treatment. As in the previous experiment [13], at least 48 h were left between each treatment.

### Behavioural Analysis

Behavioural analysis was performed according to previously published methods [16], [18], [19]. Parkinsonian disability scores were rated for 5 min every 10 min. The following items were rated: range of movement (0–9), bradykinesia (0–3), posture (0–1), and attention/ alertness (0–1). For each of the aforementioned items, the higher the score, the greater the disability. A global parkinsonian disability score was calculated as a combination of the aforementioned behaviours according to the following formula: (range of movement × 1) + (bradykinesia × 3) + (posture × 9) + (alertness × 9). The maximal parkinsonian disability score per 5 min observation period was 36.

L-DOPA-induced dyskinesia and psychosis-like behaviours were assessed concomitantly with parkinsonian disability. Dyskinesia were rated from 0–4. Choreiform and dystonic dyskinesia were rated separately and the score given reflected the most disabling dyskinesis observed, either chorea or dystonia, for every 5 min period of evaluation. Psychosis-like behaviours were also rated on a 0–4 scale. The following behaviours were scored: hyperkinesia, response to non-apparent stimuli (hallucinatory behaviour), repetitive grooming, and stereotypies [17], [18], [20]. The psychosis-like behaviour score attributed for a 5 min observation period was the most disabling of any of the four items assessed. For each of chorea, dystonia, and psychosis-like behaviours, the higher the score, the greater the disability.

Scores were cumulated for each hour across the entire 6 h of observations and during the peak-effect period (80–140 min following L-DOPA administration). Duration of anti-parkinsonian action, *i.e.* ON-time, was defined as the number of minutes for which the bradykinesia score was 0. ON-time was further divided as “good” or “bad” quality, depending on the severity of dyskinesia present. “Good quality” ON-time was defined as the number of minutes during which dyskinesia were either absent, mild, or moderate in intensity (0–2), while “bad quality” ON-time was defined as the number of minutes during which dyskinesia were either marked or severe (3–4).

### Statistical Analysis

Categorical, discontinuous scores for parkinsonian disability, dyskinesia and psychosis-like behaviours severity were analysed using non-parametric Friedman’s followed by Dunn’s multiple comparison *post hoc* tests. Continuous ON-time parameters were analysed by one-way repeated measure analysis of variance (RM ANOVA) followed by Tukey’s or Dunnett’s multiple comparison *post hoc* tests. Time course data for parkinsonian disability and dyskinesia scores were ranked by animal across each of the four treatments and analysed by a two-way ANOVA followed by Bonferroni’s multiple comparison *post hoc* tests. Statistical significance was assigned when *P*<0.05. Analyses were performed using GraphPad Prism 5.03 (GraphPad Software, La Jolla, USA) and Microsoft Office Excel 2007 (Microsoft Corporation, Redmond, USA).

## Results

### Effects of UWA-101 on L-DOPA Anti-parkinsonian Action in the MPTP-lesioned Common Marmoset

**Figure 2:**
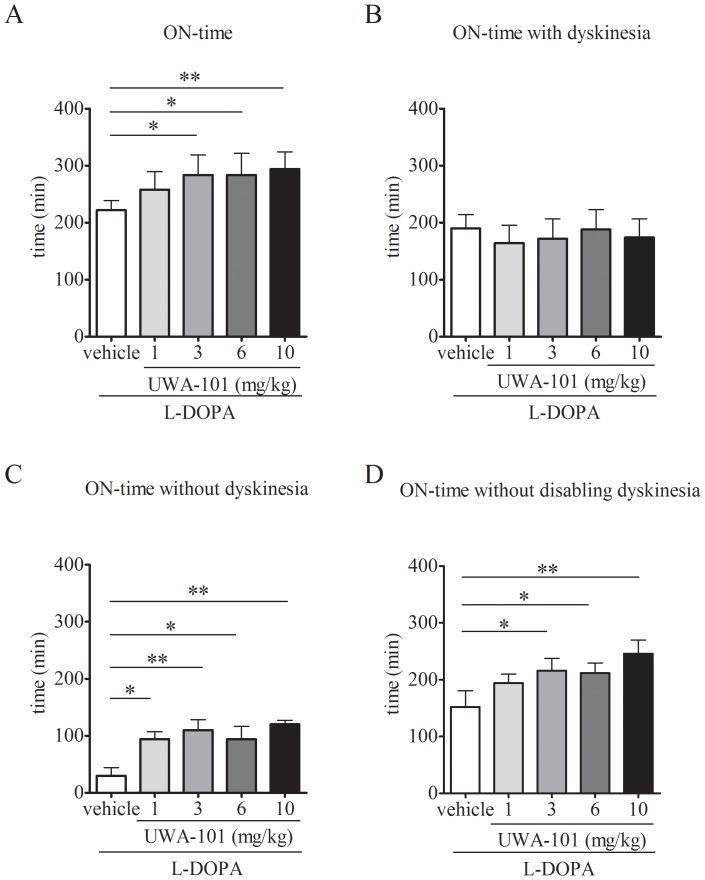
ON-time and quality of ON-time. A. UWA-101 (3, 6, 10 mg/kg), when co-administered with L-DOPA, significantly increased duration of ON-time compared to L-DOPA/ vehicle treatment. Following administration of L-DOPA/ vehicle, marmosets had a mean duration of ON-time of 221.8±19.0 min. Combining L-DOPA with UWA-101 3 or 6 mg/kg both led to an additional 62 min of ON-time, while UWA-101 10 mg/kg led to an additional 72.2 min of ON-time (all *P*<0.05). B. UWA-101 (1, 3, 6, 10 mg/kg), in combination with L-DOPA, did not alter duration of ON-time with dyskinesia. Following administration of L-DOPA/ vehicle, the mean duration of ON-time with dyskinesia was 190.0±26.9 min. This was not significantly modified when UWA-101 was added to L-DOPA (all *P*>0.05). C. UWA-101 (1, 3, 6, 10 mg/kg), in combination with L-DOPA, significantly increased duration of ON-time without dyskinesia. The administration of L-DOPA/ vehicle led to a mean duration of ON-time without dyskinesia of 30.0±15.8 min. When co-administered with L-DOPA, UWA-101 (1, 3, 6, 10 mg/kg) , increased duration of ON-time without dyskinesia by 64 min, 80 min, 64 min and 90 min, respectively (*P*<0.05 for UWA-101 1 and 6 mg/kg, and *P*<0.01 for UWA-101 3 and 10 mg/kg). D. UWA-101 (10 mg/kg) significantly extended duration of ON-time without disabling dyskinesia, when combined with L-DOPA. Following L-DOPA/ vehicle treatment, duration of ON-time without disabling dyskinesia was 152.0±32.1 min. The co-administration of UWA-101 10 mg/kg added 94 min (*P*<0.01). *: *P*<0.05; **: *P*<0.01. Data are expressed as the mean ± SEM.

Following the administration of L-DOPA/ vehicle, ON-time duration was 221.8±19.0 min. Co-administration of UWA-101 (3, 6 and 10 mg/kg) with L-DOPA resulted in significant increases in duration of ON-time (*F*(4,16) = 6.569, *P*<0.01, one-way RM ANOVA). Thus, mean ON-time duration was 283.8±39.0 min following L-DOPA/ UWA-101 3 mg/kg treatment (28% increase, *P*<0.05, Tukey’s *post hoc* test), 283.8±42.7 min following L-DOPA/ UWA-101 6 mg/kg treatment (28% increase, *P*<0.05, Tukey’s *post hoc* test) and 294.0±33.8 min following L-DOPA/ UWA-101 10 mg/kg treatment (33% increase, *P*<0.01, Tukey’s *post hoc* test, Figure 2A).

The increase in total ON-time duration was not due to an increase in ON-time with dyskinesia. Thus, mean ON-time with dyskinesia duration was 190.0±26.9 min in the L-DOPA/ vehicle group and was not significantly altered by adding UWA-101, regardless of the dose (*F*(4,16) = 0.4745, *P*>0.05, one-way RM ANOVA, Figure 2B).

However, the addition of UWA-101 (1, 3, 6 and 10 mg/kg) to L-DOPA resulted in a significant increase in duration of ON-time without dyskinesia (*F*(4,16) = 5.33, *P*<0.01, one-way RM ANOVA). Mean duration of ON-time without dyskinesia was 30.0±15.8 min in the L-DOPA/ vehicle group, while it reached 94.0±14.8 min in the L-DOPA/ UWA-101 1 mg/kg group (213% increase, *P*<0.05, Dunnett’s *post hoc* test), 110.0±20.3 min in the L-DOPA/ UWA-101 3 mg/kg group (267% increase, *P*<0.01, Dunnett’s *post hoc* test), 94.0±24.9 min in the L-DOPA/ UWA-101 6 mg/kg group (213% increase, *P*<0.05, Dunnett’s *post hoc* test) and 120.0±7.9 in the L-DOPA/ UWA-101 10 mg/kg group (300% increase, *P*<0.01, Dunnett’s *post hoc* test, Figure 2C).

UWA-101 (10 mg/kg) also extended duration of ON-time without disabling dyskinesia, *i.e*. “good” ON-time (*F*(4,16) = 4.146, *P*<0.05, one-way RM ANOVA, Figure 2D). Thus, duration of ON-time without disabling dyskinesia was 152.0±32.1 in the L-DOPA/ vehicle group and 246.0±26.6 in the L-DOPA/ UWA-101 10 mg/kg group (62% increase, *P*<0.01, Tukey’s *post hoc* test).

### Effects of UWA-101 on L-DOPA-induced Dyskinesia in the MPTP-lesioned Common Marmoset

**Figure 3:**
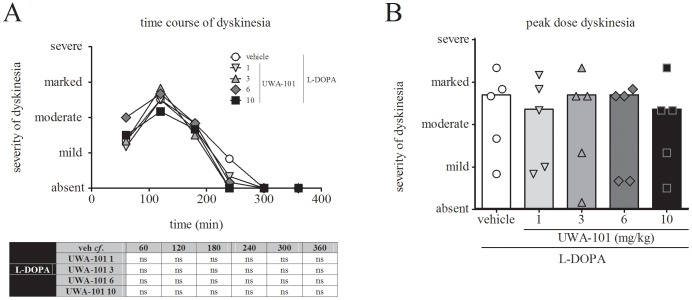
Dyskinesia. A. Dyskinesia time course. At no time during the 6 h observation period did UWA-101 (1, 3, 6, 10 mg/kg) exacerbate the severity of dyskinesia when compared to L-DOPA/ vehicle treatment (*P*>0.05). Each time point represents the cumulated dyskinesia scores for every 5 min observation period during the preceding 60 min. The maximal possible score (most severe disability) was 24. On the *y*-axis, mild = 6, moderate = 12, marked = 18, severe = 24. B. Peak dose dyskinesia. UWA-101 (1, 3, 6, 10 mg/kg) in combination with L-DOPA did not exacerbate the severity of peak dose dyskinesia (sum of dyskinesia score for every 5 min observation period from 80–140 min following treatment, during which dyskinesia severity was maximal) when compared to L-DOPA/ vehicle treatment (*P*>0.05). Median peak dose dyskinesia severity was moderate – marked in each treatment group. The maximal possible score (most severe disability) was 24. On the *y*-axis, mild = 6, moderate = 12, marked = 18, severe = 24. Data are expressed as the median (A) and as the median with individual scores (B). ns: not significant.

Co-administration of UWA-101 (1, 3, 6 and 10 mg/kg) with L-DOPA did not exacerbate the severity of L-DOPA-induced dyskinesia over the time course of the assessment (*F*_time_(5,120) = 0.0, *P*>0.05, *F*_treatment_(4,120) = 0.7338, *P*>0.05, and *F*_interaction_(20,120) = 0.7268, *P*>0.05, two-way ANOVA), when compared to L-DOPA/ vehicle treatment (Figure 3A). UWA-101 (1, 3, 6 and 10 mg/kg) did not exacerbate peak dose dyskinesia (Friedman statistics = 4.909, *P*>0.05, Friedman’s test), when compared to L-DOPA/ vehicle treatment (Figure 3B).

### Effects of UWA-101 on L-DOPA-induced Psychosis-like Behaviours in the MPTP-Lesioned Common Marmoset

**Figure 4:**
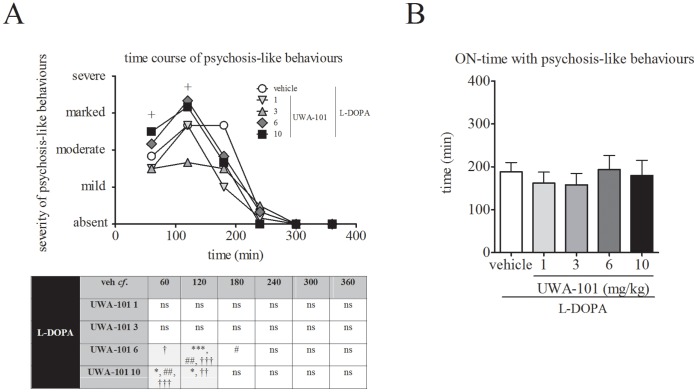
Psychosis-like behaviours. A. UWA-101 (6, 10 mg/kg) exacerbated the severity of psychosis-like behaviours. The addition of UWA-101 6 mg/kg to L-DOPA resulted in significantly more severe psychosis-like behaviours when compared to L-DOPA/ vehicle (at time 120 min post drug administration, *P*<0.001). L-DOPA/ UWA-101 6 mg/kg also led to significantly more severe psychosis-like behaviours than the L-DOPA/ UWA-101 1 mg/kg (120 min and 180 min post drug administration, *P*<0.01 and *P*<0.05, respectively) and the L-DOPA/ UWA-101 3 mg/kg (60 min and 120 min post drug administration, *P*<0.05 and *P*<0.001, respectively) treatments. Psychosis-like behaviours were also significantly more severe when L-DOPA/ UWA-101 10 mg/kg was compared to L-DOPA/ vehicle (60 and 120 min post drug administration, both *P*<0.05), L-DOPA/ UWA-101 1 mg/kg (60 min post drug administration, *P*<0.01) and L-DOPA/ UWA-101 3 mg/kg (60 min and 120 min post drug administration, *P*<0.001 and *P*<0.01, respectively) treatments. Each time point represents the cumulated psychosis-like behaviours scores for every 5 min observation period during the preceding 60 min. The maximal possible score (most severe disability) was 24. On the *y*-axis, mild = 6, moderate = 12, marked = 18, severe = 24. The crosses on the graph represent time points for which there is statistical significance. B. UWA-101, in combination with L-DOPA, did not alter duration of ON-time with psychosis-like behaviours. Following administration of L-DOPA/ vehicle, the mean duration of ON-time with psychosis-like behaviours was 188.0±24.8 min. This was not significantly modified when UWA-101 (1, 3, 6, 10 mg/kg) was added to L-DOPA (all *P*>0.05). *: *P*<0.05 when compared to vehicle; ***: *P*<0.001 when compared to vehicle; #: *P*<0.05 when compared to UWA-101 1 mg/kg; ##: *P*<0.01 when compared to UWA-101 1 mg/kg; †: *P*<0.05 when compared to UWA-101 3 mg/kg; ††: *P*<0.01 when compared to UWA-101 3 mg/kg; †††: *P*<0.001 when compared to UWA-101 3 mg/kg. Data are expressed as the median (B) and as the mean ± SEM (B).

Co-administration of UWA-101 (6 and 10 mg/kg) with L-DOPA significantly increased the severity of psychosis-like behaviours when compared to L-DOPA/ vehicle treatment (*F*_time_(5,120) = 0.0, *P* = 1.00, *F*_treatment_(4,120) = 8.954, *P*<0.001, and *F*_interaction_(20,120) = 2.152 *P*<0.05, two-way ANOVA, Figure 4A). Co-administration of lower doses of UWA-101 (1 and 3 mg/kg) with L-DOPA had no effect on psychosis-like behaviours severity when compared to L-DOPA/ vehicle (*P*>0.05 for both, Bonferroni’s *post hoc* test). Psychosis-like behaviours following L-DOPA/ UWA-101 treatment were significantly more severe during the first (10 mg/kg, *P*<0.05, Bonferroni’s *post hoc* test) and second hour of observation (6 and 10 mg/kg, *P*<0.001 and *P*<0.05, respectively, Bonferroni’s *post hoc* test) when compared to the L-DOPA/ vehicle treatment.

**Table 1:** Order of treatments.

study day	animal 1	animal 2	animal 3	animal 4	animal 5
**1**	3 mg/kg	vehicle	1 mg/kg	3 mg/kg	vehicle
**2**	vehicle	6 mg/kg	10 mg/kg	vehicle	3 mg/kg
**3**	6 mg/kg	3 mg/kg	vehicle	6 mg/kg	6 mg/kg
**4**	10 mg/kg	1 mg/kg	3 mg/kg	10 mg/kg	10 mg/kg
**5**	1 mg/kg	10 mg/kg	6 mg/kg	1 mg/kg	1 mg/kg

Table 1 shows the randomised Latin square design employed in the current study. Such a design allows all subjects to receive all treatments, once, in a different, randomly assigned, order, thereby minimising the risk of carryover effect.

Co-administration of UWA-101 with L-DOPA did not increase duration of ON-time with psychosis-like behaviours (Figure 4B). Thus, mean duration of ON-time with psychosis-like behaviours was 188.0±24.8 min in the L-DOPA/ vehicle group, and this was not significantly modified following the addition of UWA-101, regardless of the dose (*F*(4,16) = 1.987, *P*>0.05, one-way RM ANOVA).

## Discussion

The present study expands previous work with UWA-101 performed by our group, using a wider range of UWA-101 doses, a different group of marmosets and a longer observation period, allowing the assessment of the effect of UWA-101 on duration of ON-time. We demonstrate that co-administration of the DAT/ SERT inhibitor UWA-101 with L-DOPA can extend total ON-time duration. At no time did UWA-101 increase the severity of dyskinesia. UWA-101 thus increased the duration of ON-time without disabling dyskinesia. However, higher doses of UWA-101 led to an increase in the severity of L-DOPA-induced psychosis-like behaviours. These data confirm and extend a previous report on the actions of UWA-101 in MPTP-lesioned non-human primates [13].

### UWA-101 does not Exacerbate L-DOPA-induced Dyskinesia

The actions of UWA-101 in extending total duration of ON-time are similar to those of its close structural analogue, and dual SERT/ DAT inhibitor, S-3,4-methylenedioxymethamphetamine (S-MDMA). However, in contrast to UWA-101, when S-MDMA was administered to MPTP-lesioned marmosets, it exacerbated dyskinesia severity [21]. UWA-101 is essentially equipotent in inhibiting DAT and SERT [13], while S-MDMA is a SERT>DAT inhibitor, with a 10∶1 ratio [21]. Thus, while it seems clear that dual SERT/ DAT inhibition extends duration of L-DOPA anti-parkinsonian efficacy, the SERT/ DAT ratio appears critical in determining the quality of the extra ON-time. In the case of S-MDMA however, reversal of SERT and DAT gradient [22], thus increasing the synaptic concentrations of dopamine, might also have contributed to exacerbating dyskinesia severity.

The reason why a balanced, in contrast to a SERT>DAT, inhibitor may not exacerbate dyskinesia can only be speculated upon. While difficult to define theoretically, it is not hard to imagine that there might be a “sweet spot” of relative affinities for DAT and SERT that will maximise the ability of DAT/ SERT inhibitors to increase physiological dopamine transmission without increasing non-physiological transmission. The following discussion will focus on aberrant dopamine release by raphestriatal serotonergic axons and dopamine release by the remaining nigrostriatal fibres, though an involvement at other sites is possible.

Serotonergic raphestriatal terminals have been suggested to be one site involved in the pathophysiology of L-DOPA-induced dyskinesia, as raphestriatal terminals can metabolise L-DOPA into dopamine [23]–[25] and release it, as a “false neurotransmitter”, in the striatum. The overspill of dopamine to nigrostriatal synapses is likely responsible for the enhancement of anti-parkinsonian benefits of L-DOPA. Raphestriatal terminals will also, via SERT, participate in dopamine re-uptake [26], [27]. Inhibition of SERT, by UWA-101 or S-MDMA, will enhance this overspill and thus enhance anti-parkinsonian benefits of L-DOPA. Inhibition of DAT, by UWA-101 or S-MDMA, in surviving terminals of the damaged nigrostriatal pathway will increase the possibility of interaction of dopamine with its receptors at the nigrostriatal synapse and thus further contribute to the enhancement of L-DOPA anti-parkinsonian benefits.

However, because raphestriatal terminals lack the autoregulatory mechanisms proper to dopaminergic transmission, raphestriatal dopamine is released in a non-physiological manner, which is thought to lead to dyskinesia [28]–[30]. For the same reasons that SERT inhibition could lead to an increased availability of dopamine at nigrostriatal synapses, and contribute to anti-parkinsonian benefit, it would also exacerbate of the non-physiological raphestriatal L-DOPA-derived dopaminergic transmission, and exacerbate dyskinesia.

The balanced inhibition of SERT and DAT by UWA-101 may provide a means of enhancing the availability of dopamine at nigrostriatal synapses without greatly enhancing dopamine levels at raphestriatal synapses. The SERT-predominant actions of S-MDMA may shift the balance further in terms of non-physiological dopaminergic transmission and thereby exacerbate dyskinesia. While SERT inhibition will, as described above, increase dopamine levels, in the context of L-DOPA-treated parkinsonian animals, it will also increase serotonin (5-HT) levels. SERT inhibition will thus lead to 5-HT-mediated activation of serotonergic type 1A (5-HT_1A_) receptors, presynaptic autoreceptors which, once activated, reduce striatal dopamine release from raphestriatal terminals [31], [32]. The combined action of DAT and SERT inhibition could thus lead to more physiological dopamine signalling than inhibition of either alone; indeed keeping this in balance, as opposed to inhibiting SERT more than DAT, could be one further mechanism explaining why UWA-101, unlike S-MDMA, did not exacerbate dyskinesia severity. Moreover, dopamine itself could participate in this 5-HT_1A_-mediated regulatory process, as it is a low-affinity partial agonist at 5-HT_1A_ receptors [33].

### UWA-101 Exacerbates the Severity of Psychosis-like Behaviour at High Doses

In a previous series of experiments, we demonstrated that UWA-101 did not induce behaviours attributable to psychoactivity when administered as monotherapy to normal, non-parkinsonian animals [13]. However, in the current study, adding UWA-101 to L-DOPA resulted in an exacerbation of L-DOPA-induced psychosis-like behaviours at the higher doses examined.

We hypothesise that these effects to exacerbate psychosis emerge from inhibition of SERT and indirect stimulation of serotonergic type 2A (5-HT_2A_) receptors. Thus, as described above, in the presence of L-DOPA, inhibition of SERT by UWA-101 would lead to increased dopamine levels surrounding serotonergic synapses. Dopamine binds to 5-HT_2A_ receptors, at which it acts as a partial agonist [34]. 5-HT_2A_ receptors are believed to be important in the genesis of psychotic symptoms in PD and other disorders. Indeed, activating 5-HT_2A_ receptors is thought to be an important mechanism of action of hallucinogens [35] and, conversely, antagonising 5-HT_2A_ receptors is believed to underlie the action of atypical antipsychotics such as clozapine [36], [37]. Moreover, 5-HT_2A_ receptors are increased in the temporal cortex of PD patients with visual hallucinations [38], [39]. Thus, UWA-101, by blocking both SERT and DAT would elevate dopamine levels and thereby increasing stimulation, by dopamine, of 5-HT_2A_ receptors.

### Concluding Remarks

Since UWA-101 is the first dual SERT/ DAT inhibitor to be tested in PD models, it is hard to generalise but, we propose that, in order to employ monoamine re-uptake inhibitors, in combination with L-DOPA, to increase duration of ON-time without adversely affecting dyskinesia, it might be necessary to antagonise both the SERT and DAT. However, it will be necessary to define optimal therapeutic windows with this class of drugs because at higher doses it appears that dual SERT/ DAT inhibition can exacerbate the severity of psychosis-like behaviours. Moreover, whether the anti-parkinsonian efficacy and the lack of deleterious effect on dyskinesia severity, of UWA-101 as adjunct therapy to L-DOPA will be maintained after chronic administration remains unknown.
